# Laparoscopic versus Open Total Gastrectomy for Locally Advanced Gastric Cancer: Short and Long-Term Results

**DOI:** 10.3390/curroncol29110665

**Published:** 2022-11-06

**Authors:** Sara Di Carlo, Leandro Siragusa, Alessia Fassari, Enrico Fiori, Francesca La Rovere, Paolo Izzo, Valeria Usai, Giuseppe Cavallaro, Marzia Franceschilli, Sirvjo Dhimolea, Simone Sibio

**Affiliations:** 1 Department of Surgical Sciences, University of Rome “Tor Vergata”, Viale Oxford 81, 00133 Rome, Italy; 2 Department of Surgery “Pietro Valdoni”, Sapienza University of Rome, “Umberto I” University Hospital, Viale del Policlinico 155, 00161 Rome, Italy

**Keywords:** gastric cancer, laparoscopic gastrectomy, open gastrectomy, D2 lymphadenectomy

## Abstract

Background: Laparoscopic gastrectomy for early gastric cancer is widely accepted and routinely performed. However, it is still debated whether the laparoscopic approach is a valid alternative to open gastrectomy in advanced gastric cancer (AGC). The aim of this study is to compare short-and long-term outcomes of laparoscopic (LG) and open (OG) total gastrectomy with D2 lymphadenectomy in patients with AGC. Methods: A retrospective comparative study was conducted on patients who underwent LG and OG for ACG between January 2015 and December 2021. Primary endpoints were the following: recurrence rate, 3-year disease-free survival, 3-year and 5-year overall survival. Univariate and multivariate analysis was conducted to compare variables influencing outcomes and survival. Results: Ninety-two patients included: fifty-three OG and thirty-nine LG. No difference in morbidity and mortality. LG was associated with lower recurrence rates (OG 22.6% versus LG 12.8%, *p* = 0.048). No differences in 3-year and 5-year overall survival; 3-year disease-free survival was improved in the LG group on the univariate analysis but not after the multivariate one. LG was associated with longer operative time, lower blood loss and shorter hospital stay. Lymph node yield was higher in LG. Conclusion: LG for AGC seems to provide satisfactory clinical and oncological outcomes in medium volume centers, improved postoperative results and possibly lower recurrence rates.

## 1. Introduction

Gastric cancer (GC) is the fifth most widely spread cancer and the third leading cause of cancer-related death worldwide, although its incidence has decreased over the past decade [1,2]. Despite the rapid development of radiotherapy, chemotherapy and immunotherapy, surgical resection with adequate lymphadenectomy represents the gold standard treatment option with curative intent for early and some advanced forms of gastric cancer [3,4,5,6,7].

Multimodality treatment, neoadjuvant chemotherapy, R0 surgical resection and adequate lymphadenectomy, postoperative adjuvant chemotherapy and radiotherapy appear to improve the survival and disease-free survival of patients with gastric cancer [8,9].

Since Kitano et al. first performed a laparoscopic distal gastrectomy for early gastric cancer (EGC) in 1994, this minimally invasive approach has progressively spread worldwide [10]. The main advantages of laparoscopic surgery over conventional open surgery are: a smaller incision, reduction in surgical stress, less blood loss, quicker return to normal bowel function, earlier resumption of oral intake, less postoperative pain and faster recovery [11,12,13,14,15].

Another benefit of laparoscopy is the amplification of the view leading to a more meticulous lymph node dissection, which is an important factor for the patient’s prognosis [16]. Nevertheless, a laparoscopic D2 lymphadenectomy in AGC is considered more difficult to perform due to several limitations such as a reduced degree of freedom, possible tearing of the soft tissue and unsecure bleeding control.

Therefore, advanced surgical experience is crucial to perform safely these challenging upper gastrointestinal procedures [17,18,19,20].

Prospective studies have confirmed the safety and feasibility of the laparoscopic approach for early gastric cancer, and it is recommended as a standard treatment in Japan and South Korea, but the adequate approach of advanced gastric cancer still needs to be validated [12,21,22].

The use of minimally invasive surgery to treat advanced stage tumors remains highly controversial, mainly because of concerns related to the adequacy of the surgical resection and the possibility of performing an adequate lymph node dissection [23,24,25,26,27,28]. Despite technological advances, the laparoscopic approach is a challenging and time-consuming procedure and involves a steep learning curve [29,30].

While several randomized control trials (RCTs) and meta-analyses have been published for EGC patients, only a few studies have been conducted for AGC, which are based on a smaller scale and are predominantly retrospective [31,32].

All the randomized clinical trials were conducted at high volume centers such as Japan, Korea and China, where the results tend to be superior [33]. The CLASS-01 study in China [34], the JLSSG 0901 study in Japan [17] and the KLASS-02 in Korea [33] all showed comparable postoperative morbidity as well as survival rates for laparoscopic distal gastrectomy (LDG) and open distal gastrectomy.

Currently, a laparoscopic gastrectomy (LG) with D2 lymphadenectomy is safely performed only in high volume centers in Japan and South Korea [23].

Considering the controversy over the laparoscopic approach for AGC, the aim of this retrospective study was to evaluate short- and long-term outcomes of laparoscopic gastrectomy (LG) versus open gastrectomy (OG) in patients with AGC over a six-year period in medium volume centers.

## 2. Materials and Methods

A retrospective comparative study of all patients meeting inclusion criteria who underwent LG and OG for ACG between January 2015 and December 2021 was conducted.

Patients were divided into two groups according to surgical approach: open and laparoscopic.

During the study period, all patients with preoperative diagnosis of non-metastatic, locally advanced gastric adenocarcinoma (Stage II–III) seen at our institutions and eligible for potentially curative D2 gastrectomy were included. Data on short- and long-term outcomes were prospectively collected and reviewed. Exclusion criteria included age below 18, palliative surgery, GIST or neuroendocrine tumor, intraoperative evidence of distant metastasis or peritoneal carcinosis.

### 2.1. Preoperative Assessment of Patients

All patients had the same diagnostic work-up according to a standard protocol (endoscopy with biopsy, abdominal computed tomography, endoscopic ultrasound) and were managed in a multidisciplinary setting (MDT). Until 2017, neoadjuvant chemotherapy based on the MAGIC regimen was offered to patients fit enough to cope with multi-modal treatment [35]. From 2017, the FLOT regimen was offered to the same category of patients, given the fact that this chemotherapy system demonstrated improved progression-free and overall survival rates, and showed more frequent pathologic complete regression [36].

After re-staging investigations on patients treated with chemotherapy, the resectability of the gastric cancer was re-assessed during MDT meetings. Patients with bleeding or obstruction typically forego neoadjuvant treatment and proceed straight to surgery and subsequently received an extra cycle of chemotherapy adjuvant.

### 2.2. Surgical Technique

Both laparoscopic and open surgery with D2 lymphadenectomy were performed by the same experienced upper gastrointestinal surgeons.

A fully laparoscopic approach with side-to-side intracorporeal anastomosis and Roux-en-Y reconstruction was adopted for LG. Open resections (OG) were performed through a midline incision. Modalities of lymph node harvesting were the same for both the open and laparoscopic approach. Lymph node dissection was conducted preferably en bloc, but this was not always possible for station 8, 9, 11. In both cases, the reconstruction technique included a retrocolic or antecolic Roux-en-y oesophagojejunostomy.

### 2.3. Endpoints

Primary endpoints were: recurrence, 3-year disease-free survival (DFS), 3-year overall survival and 5-year overall survival rates (OS). Secondary endpoints were: mortality, intraoperative complication rate, postoperative complication rate, surgical time (min), length of postoperative hospital stay (day), estimated blood loss, time to first liquid diet, number of retrieved lymph nodes and adequacy of lymphadenectomy (number of lymph nodes >15). Any complication (understood as any adverse event occurring during the 30-day postoperative period) including anastomotic leak, abdominal collection, bleeding, pulmonary complications (clinical symptoms, confirmed by radiological examination), pancreatitis, delayed gastric emptying or surgical site infection (SSI, defined according to the Centre for Disease Control and Prevention, CDC/NHNS) was recorded and graded according to Clavien–Dindo classification [37,38].

### 2.4. Follow-Up and Survival

This research only included patients who underwent an adequate follow-up. Recurrence was classified as either local or distant, depending on the first recognized disease site. We considered survival time as the time from the date of the operation until death or the last available follow-up. After surgery, the patients were followed regularly with the same protocol, and data including recurrence and death were recorded. Follow-up was conducted every three months for the first two years postoperatively and every six months for the next three years. Follow-up care included medical history every three months, physical examination and blood test every three months, CT scan every six months for three years and upper gastrointestinal endoscopy annually for three years. In patients without symptoms, recurrence was detected on regular follow-up investigations such as abdominal computed tomography (CT).

### 2.5. Statistical Analysis

Data were recorded as number and percentages. Univariate analysis concerning binomial variables was performed by means of chi square test or Fisher’s exact test. Comparison of continuous variables was performed with the independent samples t-test. Independent variables found significant at univariate analysis were matched in multivariate analysis conducted by means of the Cox proportional hazard regression model to determine their impact on survival between the two groups (open or laparoscopic). Overall survival was plotted in Kaplan–Meier curves and a log-rank test was used for the comparison of the distributions between the two groups. A *p* value less than 0.05 was considered significant.

### 2.6. Ethics

This study was conducted according to the international ethical recommendations on clinical research established by the Helsinki Declaration. The study was conducted in accordance with STROBE criteria (https://strobe-statement.org, accessed on 2 August 2022) [39].

## 3. Results

From January 2015 to December 2021, ninety-nine consecutive patients diagnosed with advanced gastric cancer were scheduled for D2 total gastrectomy. Four patients who had histopathological diagnosis of gastrointestinal stromal tumors (GISTs) and three with intraoperative finding of peritoneal carcinosis were excluded from the analysis. Out of the remaining 92 patients, 53 (57.6%) underwent OG and 39 (42.4%) LG.

Baselines patients’ characteristics are summarized in Table 1.

The two groups were homogenous with respect to age, sex, BMI, American Society of Anesthesiologists (ASA) score, clinical T- and N-stage, and the proportion of patients receiving perioperative chemotherapy. 85% of patients in the open group and 77% in the laparoscopic group had clinical T3/T4 cancers while 58.5% and 51.3% of patients, respectively, had positive lymph nodes.

Operative outcomes are summarized in Table 2.

There was no conversion to open surgery. Although the operating time was significantly longer in the LG, the laparoscopic approach provided a significantly shorter length of stay (LOS) and total hospital stay (that included readmissions within 30 days of surgery) as well as lower blood loss and time to first oral intake (clear fluids). There were no differences either in intraoperative complications, rates of overall complications, readmissions, reoperations or mortality. Minor complications (CD I-II-IIIa) occurred in four patients in the open group and in five in the laparoscopic group. Major complications requiring reoperation occurred in four patients in the open group (bleeding in two patients, anastomotic leakage in one patient and postoperative bowel obstruction in another patient) and in two patients in the laparoscopic group (one anastomotic leakage treated with laparoscopic wash-out and drainage and endoscopic insertion of stent and one internal hernia). One patient in the open group required ICU admission for acute renal failure and respiratory distress. One patient in the open group and one in the laparoscopic group, respectively, required reoperation for anastomotic leakage and died of sepsis after readmission to the ICU. However, differences in the two groups did not reach statistical significance.

Oncological outcomes are summarized in Table 3.

A higher number of patients in the OG group received adjuvant chemotherapy compared with the LG group (79.9% vs. 65.3%) The percentage of R0 resections was similar between the two groups (OG 98.1% versus LG 100%, *p* = 1.000), whereas a higher mean number of resected lymph nodes was recorded in the laparoscopic group (LG 18.7% vs. OG 15.1%, *p* = 0.031).

Additionally, the laparoscopic approach seemed to lower inadequate lymphadenectomy rates (OG 15.1% vs. LG 7.7%, *p* < 0.04). Furthermore, it was associated with significantly lower recurrence rates (OG 22.6% versus LG 12.8%, *p* = 0.048) compared to open surgery.

**Figure 1:**
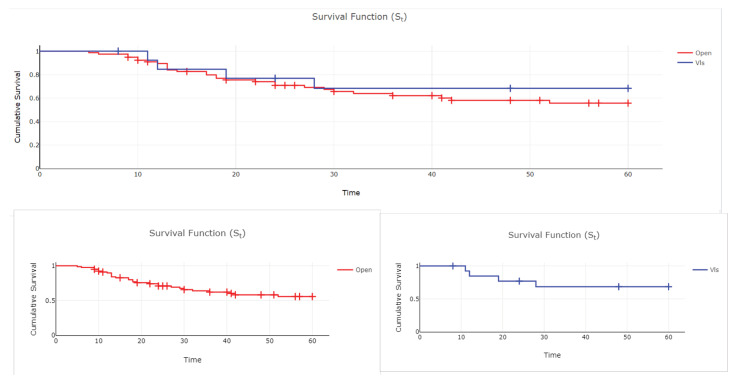
Kaplan-Meier overall survival curve according to surgical technique *p* = 0.490 (log rank test) (Color version of figure is available online).

There were no statistically significant differences in overall survival between the two groups (Figure 1).

**Figure 2:**
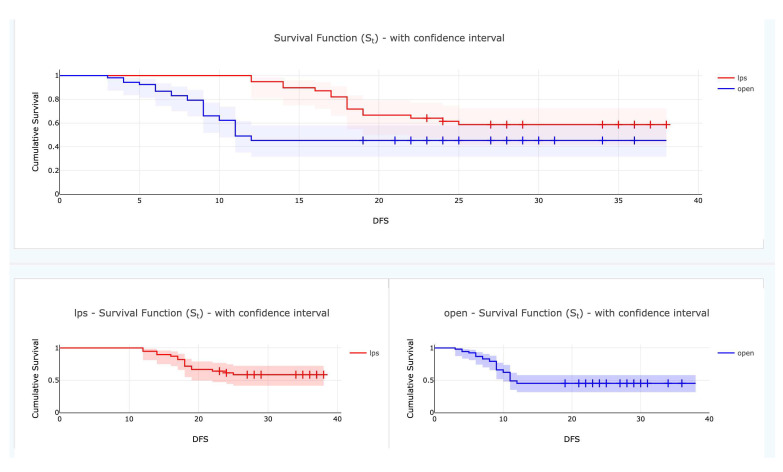
Kaplan-Meier 3-yrs disease free survival curve according to surgical technique *p* = 0.041 (log rank test) (Color version of figure is available online).

Three years of disease-free survival was improved in the laparoscopic group (*p* = 0.041) (Figure 2).

Multivariate analysis by the Cox proportional hazard regression model adjusted for the variables found significant or near significant at univariate analysis (morbidity, number of retrieved lymph nodes, recurrence rate) together with demographic characteristics (age, ASA score, AJCC stage) indicated a decreased mortality in the laparoscopic group, but this difference did not reach statistical significance (*p* value = 0.97) (Table 4).

## 4. Discussion

This comparative study confirmed that a D2 laparoscopic gastrectomy shows similar surgical (same rates of morbidity and mortality) and oncological results (same rates of R0 resections) if compared to open gastrectomy, and it is a safe and effective procedure. In accordance with previous studies, there was a trend towards improved overall survival [39,40,41,42]. Moreover, this study showed that the laparoscopic approach offered advantages over open surgery in terms of a higher number of lymph nodes retrieved, adequacy rates of lymphadenectomy, reduction of hospital stays and less blood loss despite the longer operative time. This concept was reiterated by Basil J. Ammori et al. [43], who underlines the lack of differences in operative and oncological outcomes between laparoscopic and open surgery and concluded that, although laparoscopic surgery requires a longer operative time (393 vs. 218 min), it results in less blood loss (100 vs. 200 mL) and a shorter hospital stay (3.0 days versus 7.5 days).

Chevallay et al. [44] in 2019 conducted a systematic review of European studies on this topic and found no mean difference in the number of lymph nodes harvested, no difference in short-term or long-term mortality between laparoscopic and open surgery and longer operative times in the laparoscopic approach, but a lower reoperation rate than the open group. In our series, there were no differences between the two groups in terms of reoperation.

Furthermore, the adequacy of the oncologic resection in the current study is demonstrated by the high number of lymph nodes retrieved in LG (mean 18.7) and the R0 resections obtained in both groups. Whether higher lymph node retrieval could improve gastric cancer staging and survival remains a possibility. A very interesting result of our analysis is that laparoscopic surgery in gastric cancer seems to lower recurrence rates. The three-year DFS rate in the LG was higher than in OG on the univariate analysis but not after matching for independent variables including the AJCC stage. Results are consistent with the findings of the previous studies and the explanation, as per the colon cancer, could be due to the impact of laparoscopy on the surgical stress response and on cytokine release or to a higher number of lymph nodes retrieved as also demonstrated by other studies [21,45,46,47,48]. The CLASS-01 randomized clinical trial [34] has also demonstrated that the 3-year disease-free survival in the laparoscopic group was not inferior to that in open distal gastrectomy. Peri-operative complications did not differ significantly between LG and OG, and this is in line with other studies [21,32,49,50]. However, other reports show better results for the laparoscopy with a smaller number of complications in this group [40,41,51]. Our median LOS in the LG was 6.5 days and this is shorter compared to LOS reported in other RCTs after LG (mean 9.6 days) [52]. The adoption of the ERAS protocol by the laparoscopic surgeon may have played a role in this result as already demonstrated for colon cancer. In fact, a recent meta-analysis of randomized and nonrandomized trials showed that the implementation of ERAS protocols in the peri-operative care of gastric cancer significantly shortened LOS [53,54].

There is a large variety of Eastern studies comparing laparoscopic gastrectomy with the open approach (due to the higher incidence of the disease and the existence of screening programs in the Asian population) [22,33]. A comparison between these experiences and ours is somewhat difficult.

In fact, there are significant differences between Eastern and Western populations. In addition to the aforementioned older age at the time of diagnosis, which indicates that patients have more comorbidities, the disease is usually staged as advanced in Western populations due to the lack of screening protocols. Furthermore, risk factors such as alcohol consumption and obesity are widely present in the Western world, and therefore these might affect the outcome of the radical treatment of gastric cancer.

The pros and cons of the MIS approach compared to the open approach in both distal and total gastrectomy have been extensively investigated in the East.

Among patients preoperatively diagnosed with early or locally advanced distal gastric cancer, laparoscopic distal gastrectomy has been demonstrated to be safe and effective compared with open distal gastrectomy. With an increased incidence of proximal gastric cancer in recent decades, total gastrectomy has been adopted by surgeons, and laparoscopic total gastrectomy (LTG) has become the preferred option. However, the safety and feasibility of LTG have yet to be proven because even well-trained surgeons find it technically challenging [55,56,57].

Zeng et al. conducted a meta-analysis of 17 RCTs (16 were Eastern studies, while 1 was Western), published between 2002 and 2018, and involving 5204 participants undergoing total or distal gastrectomy, with the open or laparoscopic approach, for early gastric cancer and advanced gastric cancer, with the aim of evaluating primary and secondary outcomes of the two surgical approaches [58]. With regard to primary outcomes, they demonstrated no difference in the number of lymph nodes harvested during surgery, in severe complications (Clavien-Dindo up to grade III or more), in short-term recurrence (within 6 months after surgery) and long-term recurrence (beyond 6 months after surgery), short-term mortality (within 1 month after surgery) and long-term mortality (beyond 1 month after surgery). Secondary outcomes included operative time (longer in the laparoscopic approach), intraoperative blood loss (less in the laparoscopic approach), time of first flatus (shorter time in the laparoscopic approach), time of first ambulation (shorter in the laparoscopic approach), time to first oral intake (shorter in the laparoscopic approach), length of hospital stay (shorter in the laparoscopic approach), total complications during the same hospitalization or within 30 days after the operation (no differences between the two approaches), and blood transfusions (no differences in the two groups). The authors concluded that laparoscopic gastrectomy is comparable to open gastrectomy in the primary outcomes and showed some advantages in the secondary outcomes.

These findings are similar to the results obtained in high-volume studies conducted on distal gastrectomy [32,59,60,61], on total gastrectomy [62,63] and on both approaches [64,65]. Furthermore, in all these publications, the choice of the surgical technique between laparoscopic or open did not influence the long-term outcomes, such as 3- or 5-year overall survival and disease-free survival rate. In addition, the laparoscopic technique seems to decrease mortality in multivariate analysis, although this result remains only a tendency in our study without reaching statistical significance. In our opinion, these findings need to be further investigated with additional studies to confirm if there are oncological or technical reasons that can explain this tendency, once confirmed.

As argued by Beyer et al. [52], however, further well-designed randomized controlled trials are needed to evaluate the long-term outcomes, as well as the possible benefits and risks of the laparoscopic approach.

In an interesting systematic review and meta-analysis of 12 non-randomized controlled studies, Li et al. [66] evaluated the surgical and long-term outcomes of laparoscopic and open gastrectomy performed on 1651 high-risk subjects. High-risk patients were defined by having one or more of the following conditions: age > 70 years, BMI > 30 kg/m^2^, ASA (American Society of Anesthesiologists) grade > 3 and clinical T stage 4 (cT4). The results showed significantly lower estimated blood loss in the laparoscopic group than the open group, and no significant difference between the two groups in operative time and number of harvested lymph nodes. Moreover, the time to flatus, time to food intake and postoperative hospital stay were significantly shorter in the laparoscopic group than the open one. A lower overall postoperative complication rate was observed in the minimally invasive group, significantly lower incidence of surgical and non-surgical complication in the laparoscopic group and, lastly, the pooled analysis showed no significant difference in overall survival between the two operative techniques (Table 2). Hence, this study suggests that the minimally invasive approach might be safe and feasible even in the elderly and high-risk patients.

Our study highlights a significant number of lymph-nodes retrieved with the laparoscopic approach, with a reduced rate of inadequate lymphadenectomies (<15 lymph nodes retrieved): this result is controversial, since surgery has been performed by the same experienced surgeons and according to consolidated technical protocols. It can be supposed that laparoscopy, even if more difficult and challenging to perform, allows better visualization of anatomical structures with more effective resections along vessels and anatomical planes. In our study, the laparoscopic group also showed lower local recurrence and a shorter LOS (6.5 days vs. 10.5 days in the open group). However, the improvement of LOS could be due to the implementation of ERAS protocols and perioperative care. Finally, the 5-year overall survival rate in LG was higher than the OG group; this result tended toward statistical significance without reaching it. While, on one side, several advantages of laparoscopic surgery have been reported in the treatment of gastric cancer, on the other side, the most relevant studies are still limited, especially regarding locally advanced gastric cancer, which requires an extensive, and often more challenging, lymph node dissection. In the randomized controlled multicenter study, KLASS-02, laparoscopic distal gastrectomy (LDG) with D2 lymphadenectomy was compared with distal open gastrectomy (ODG). The results showed that the average number of fully recovered lymph nodes and the mortality rate at ninety days were similar in both groups. On the other hand, though, the early morbidity rate, postoperative pain and the first day of flatus and postoperative hospital stay were significantly lower after LDG [33].

Although laparoscopic surgery can be regarded as a valid choice in the treatment of gastric cancer, there are some factors that hinder its application, such as the decreased sense of touch, the long learning curve, the uncomfortable position of the surgeons, the 2-dimensional visualization and limited range of movement of laparoscopic instruments, which makes it difficult to perform a precise lymphadenectomy. A possible solution to these issues is the use of robotic systems [67,68,69,70]. However, the higher cost of the robotic approach remains an important point of debate, which has negatively affected the expansion of this technology in recent years [71,72].

Regarding the safety of laparoscopic gastrectomy, there are some compelling Western studies, although their numerosity is not higher as it is in the East. Among these, there is certainly the LOGICA trial, a multicenter randomized controlled trial published in 2021 conducted by Arjen van der Veen et al., which assesses the oncological efficacy and safety of laparoscopic gastrectomy in a Western population. Between 2015 and 2018, a total of 227 patients with resectable (cT1-4a N0-3bM0) gastric adenocarcinoma were randomly assigned to laparoscopic (n = 115) or open gastrectomy (n = 112). This study showed a median hospital stay of 7 days (interquartile range, 5–9) in both groups (*p* = 0.34). The median blood loss was less in the laparoscopic group (150 vs. 300 mL, *p* < 0.001), whereas the mean operating time was longer (216 vs. 182 min, *p* < 0.001). Furthermore, both groups did not differ regarding postoperative complications (44% vs. 42%, *p* = 0.91), in-hospital mortality (4% vs. 7%, *p* = 0.40), 30-day readmission rate (9.6% vs. 9.1%, *p* = 1.00), R0 resection rate (95% vs. 95%, *p* = 1.00), median lymph node yield (29 vs. 29 nodes, *p* = 0.49), 1-year overall survival (76% vs. 78%, *p* = 0.74) and global health-related quality of life up to 1 year postoperatively (mean differences between + 1.5 and + 3.6 on a 1–100 scale; 95% CIs include zero). Therefore, the authors concluded that laparoscopic gastrectomy did not lead to a shorter hospital stay, while postoperative complications and oncological efficacy did not differ between the two approaches [73].

A future perspective is represented by lymph node ratio (ratio of the numbers of the metastatic lymph nodes to those of the dissected lymph nodes), also known as lymph node metastasis density. The correlation of number of lymph node metastases to lymph node ratio is obvious, but given the same number of metastases, when the number of dissected lymph nodes increases, patients seem to have a better prognosis. Many reports claim that lymph node ratio is efficient in stratifying patients’ outcomes in various clinical aspects [74]. However, the role of lymph node ratio as a biomarker in advanced gastric cancer still needs to be verified and validated by further dedicated studies before becoming an integrated part of treatment in the future.

The LOGICA trial’s findings confute a large variety of studies both from the Eastern and the Western world, reporting no difference in the length of stay within the comparison of the two surgical techniques. However, it confirmed and strengthened the already investigated advantages of the minimally invasive approach over open surgery, which leads to a quicker recovery of the patient, lower rate of postoperative complications, without influencing the oncological outcomes, morbidity or short-term mortality.

The limitations to the current study include its retrospective design and the small sample size, especially if compared to Eastern studies. However, the study is retrospective but from a prospectively collected database, and patients were not selected to be offered a LG. The only factor was the presence of an experienced laparoscopist in upper gastrointestinal surgery. The set-up of medium volume centers specializing in laparoscopic abdominal surgery and experienced in enhanced recovery programs seems the key factor for the successful development of such an LG program. Number of complications, including fistulas was low; however, mortality was also very low in this series, highlighting the importance of the multidisciplinary setting and expertise of highly specialized units [75,76,77].

## 5. Conclusions

Laparoscopic gastrectomy for locally advanced gastric cancer seems to provide satisfactory clinical and oncological outcomes in medium volume centers, improved postoperative results and possibly lower recurrence rates.

## Figures and Tables

**Table 1:** Baseline characteristics of the population study.

	Open (=53)	Laparoscopic (=39)	STDEV	*p*
Age (years) *	71 (88–53)	67 (85–46)	8.078	0.052
			8.159	
Sex, males: n (%)	33 (62.3)	25 (64,1)	0.481	0.103
			0.497	
BMI (kg/m^2^) *	24.5 (36.3–19)	22.6 (30.4–17)	3.871	0.487
			4.516	
ASA score *	2 (2–3)	2 (2–3)	0.596	0.406
			0.759	
Received Neoadjuvant chemotherapy n (%)	44 (83)	30 (76.9)	0.351	0.492
			0.363	
AJCC pathological stage: n (%) Stage II Stage III	23 (43.4)	18 (46.2)	0.342	0.425
	30 (56.6)	21 (53.8)	0.412	
Clinical T stage: n (%) T2 T3 T4	8 (15.1)	9 (23.1)	0.576	0.066
	15 (28.3)	24 (61.5)	0.593	
	30 (56.6)	6 (15.4)	0.792	
Clinical N stage: n (%) N0 N+	22 (41.5)	19 (48.7)	4.213	0.234
	31 (58.5)	20 (51.3)	3.125	

* Data shown represent median (range).

**Table 2:** Surgical outcomes of the two groups: univariate analysis.

	Open (=53)	Laparoscopic (=39)	STDEV	*p*
Operative time (min)	190 (420–60)	265 (435–170)	62,311	**<0.0001**
			93,862	
Intraoperative complications	0 (0)	0 (0)		1.000
Blood loss (mL)	537 (183–785)	176 (82–379)	122,981	**<0.003**
			73,214	
LOS (days)	10.5 (30–5)	6.5 (10–2)	4.612	**<0.002**
			1.992	
Reoperation: n (%)	4 (5.6)	2 (5,1)	0.222	0.5
			0.363	
Time to first oral intake (days)	7.2 (4–9)	2.7 (1–5)	4.234	**<0.002**
			1.932	
30-days Postoperative Complications: n (%)	9 (16.9)	8 (20.5)	0.469 0.468	0.187
CD grade				
I	2	1		
II	2	2		
III	4	4		
IV	-	-		
V	1	1		
90-days Postoperative Complications: n (%)	17 (21.8)	4 (28.6)	0.419	0.291
			0.487	
Mortality: n (%)	1 (1.9)	1 (2.6)		0.981

* Data shown represent median (range).

**Table 3:** Oncological results of the two groups: univariate analysis.

	Open (=53)	Laparoscopic (=39)	STDEV	*p*
Adjuvant chemotherapy	36 (67.9)	20 (51.3)	0.440 0.707	0.099
R0 resection n (%)	52 (98.1%)	39 (100%)		1.000
Lymph nodes retrieved *	15.1 (50–4)	18.7 (36–10)	4.498 14.142	**<0.031**
Lymph node count <15: n (%)	8 (15.1)	3 (7.7)	6.561 8.176	**<0.04**
Recurrence: n (%)	12 (22.6)	5 (12.8)	0.482 0.363	**<0.048**
Recurrence timing (months) *	12	15	8.921 1.414	0.464

* Data shown represent median (range).

**Table 4:** Multivariate analysis: Cox proportional hazard regression model according to surgical technique.

Hazard Ratios	Open	Laparoscopic	*p*
Hazard ratio for overall survival	0.98	0.57	0.056
Adjusted hazard ratio for overall survival	0.98	0.67	0.97
Adjusted for morbidity (CD III), number of retrieved lymph nodes, recurrence, age, ASA score, AJCC stage			

## Data Availability

All data generated or analyzed during this study are included in this published article. Dataset supporting reported results are protected and access availability must be obtained.
